# Recycling of Wastes Plastics and Tires from Automotive Industry

**DOI:** 10.3390/polym13132210

**Published:** 2021-07-03

**Authors:** Iveta Čabalová, Aleš Ház, Jozef Krilek, Tatiana Bubeníková, Ján Melicherčík, Tomáš Kuvik

**Affiliations:** 1 Department of Chemistry and Chemical Technologies, Faculty of Wood Sciences and Technology, Technical University in Zvolen, T. G. Masaryka 24, 960 53 Zvolen, Slovakia; bubenikova@tuzvo.sk; 2 Department of Wood, Pulp and Paper, Faculty of Chemical and Food Technology, Slovak University of Technology, Radlinského 9, 812 37 Bratislava, Slovakia; ales.haz@stuba.sk; 3 Department of Environmental and Forestry Machinery, Faculty of Technology, Technical University in Zvolen, T. G. Masaryka 24, 960 01 Zvolen, Slovakia; krilek@tuzvo.sk (J.K.); xmelichercikj@tuzvo.sk (J.M.); xkuvikt@tuzvo.sk (T.K.)

**Keywords:** polymers, pyrolysis, thermogravimetry, gas chromatography, plastic wastes, tire wastes, calorific value

## Abstract

Waste tires (granulate) and selected plastics from the automotive industry were evaluated by using the tertiary (pyrolysis) and quaternary (calorimetry) recovering. Pyrolysis is proving to be an environmentally friendly alternative to incineration and inefficient landfilling. Currently, the main challenges for pyrolysis of plastic waste are unavailability and inconsistent quality of feedstock, inefficient and hence costly sorting, and last but not least insufficient regulations around plastic waste management. Waste plastics and tire materials were characterized by TG/DTG analysis, Py-GC/MS analysis and calorimetry. TG analysis of the investigated materials gives the typical decomposition curves of synthetic polymers. The tested samples had the highest rate of weight loss process in the temperature range from 375 °C to 480 °C. Analytical pyrolysis of the tested polymers provided information on a wide variety of organic compounds that were released upon thermal loading of these materials without access to oxygen. Analytical pyrolysis offers valuable information on the spectrum of degradation products and their potential uses. Based on the results of calorimetry, it can be stated that the determined calorific value of selected plastic and rubber materials was ranging from 26.261 to 45.245 MJ/kg depending on the ash content and its composition.

## 1. Introduction

The world average annual production of cars and commercial vehicles is about 85–95,000,000 million. The average weight of the car is approximately 1.2 tons, which presents 102.5 million tons of refined processed material. The structure of its composition is as follows: scrap iron, plastics, tires, non-ferrous metals, glass, foam, car batteries, electrical waste, textiles and insulation materials [1].

The vehicles have a high proportion of plastics (Table 1), especially polyolefin (polypropylene-PP and polyethylene-PE), whose properties depend on the molecular weight and the degree of crystallinity [2]. Nowadays, polyolefins in economically developed countries are completely recovered in the form of waste plastics. The above mentioned plastics are used for the production of foils, board materials, electrical device covers, injection molded parts, irrigation pipes, various crates, etc. [3]. Polyvinyl chloride (PVC) and polyurethane (PU) are mass-produced synthetic plastics and are also used in the automotive industry. Their properties depend on both the production and the method of processing. PVC waste is used for sewer pipes, extruded profiles and boards production; mixed waste of PVC and polyolefin is processed for pallets and parts of floors of industrial companies [4]; and PU waste as an additive to mixtures of thermoplastic polyurethanes for increasing dimensional stability and abrasion resistance [4,5].

Nowadays, in the automotive industry, there is a growing trend to replace metals with plastic components, which leads to a reduction in the weight of cars and thus to a reduction in fuel consumption. Due to the large number of different plastic materials, the recovering of plastics brings big benefits.

The global problem of hard removable waste is related to the constantly increasing number of cars and thus to the growth of worn tires, whose primary raw material is rubber. Rubber is a raw material characterized by an unusual combination of physical properties—high elasticity with low hardness and extremely high ductility. A large number of additives is used to influence the processing behavior affect, set the required application properties and reduce material costs. Active and inactive carbon black, and dispersing additives (SiO_2_, ZnO, Al_2_O_3_, MgCO_3_) are the most important rubber fillers. The largest area (35–45%) for rubber utilization is the production of tires. Natural rubber (NR), polyisoprene rubber (IR), butadiene-styrene rubber (SBR) and polybutadiene rubber (BR) are used for their manufacturing [2].

As with plastics waste, both waste rubber and worn tires are still a global problem, and their content is still much higher than the amount of waste that can be rationally recovered. Every year, millions of tires are discarded, thrown away or buried. No regeneration process can be used to recover the original rubber or other rubber raw materials from the rubber waste. The tire contains a number of chemicals with energy potential, but it is also a source of secondary raw material. Tires consist of rubber (46–48%), carbon black (25–28%), steel inserts (10–12%), oil and vulcanizing agents (10–12%) and embedded synthetic yarns and textiles (3–6%). Tires as a secondary material are used in only two ways: material conversion (floors, noise walls, etc.) and energy recovery [11].

The aim of many scientists is to develop new methods for recycling some of the most produced plastics and rubbers. Polyolefins (PP, PE) are the main group of synthetic plastics and their wastes are very attractive material for many kinds of chemical transformation. Tertiary recycling, sometimes referred to as chemical recycling, uses chemical processes to break down polymers into value-added commodities. Typical processes include hydrolysis [12,13,14] and pyrolysis [15,16,17] of waste plastics. According to Al-Salem et al. [18] the pyrolysis of plastic wastes has gained importance due to its better environmental benefits against pollution and reduction of the carbon footprint of plastic products by minimizing carbon monoxide and carbon dioxide emission compared to combustion and gasification. Pyrolysis means the process of thermal decomposition of polymers at a temperature of 400 (450)–800 °C in a shorter time and under oxygen-free conditions [19]. During this process, carbon products are generated, such as residues and volatile hydrocarbons, which can be condensate as fuel and non-condensible as gaseous fuel [20,21]. The products of PP and PE thermal cracking are mainly a mixture of olefins (C1-C4) and aromatic compounds (benzene, toluene, xylene) [22]. The main products of polystyrenes (PS) pyrolysis is styrene. According to Wong et al. [23] and Achilias et al. [24] liquid pyrolysis products from PP are similar to crude oil, but these products show the presence of ash and wax from raw materials, which reduce the quality [25].

From a perspective point of view, rubber waste is also advantageous to use as a raw material for the chemical industry. Pyrolysis carbon, oil and gas, and steel cord are the final products of the pyrolysis processing of rubber waste (worn tires). Pyrolysis gas can be used directly in the technological process for the both heat and electricity production. It can ensure the energy self-sufficiency of the technological plant. Pyrolysis carbon-fine black powder (active coal), due to its high calorific value, can be also used as an alternative fuel. After its further processing can be used as a component of filtration equipment (in wastewater treatment plants). Additive in the rubber industry (partial replacement of carbon black, absorbent material in oil refineries, pigment in the production of plastics) is another possibility of pyrolysis carbon utilization. Pyrolysis oil, due to its high energy content, can be used directly for producing of heat or electricity. During its further processing (hydrogenation, fractional distillation, cracking) there is possible to obtain a wide range of chemicals that can be reused in various chemical industries. In tires, there are several types of steel wires, which are a secondary raw material for metallurgy. Liquefaction process of old tires is also a chemical recycling of waste rubber. It is the process of dry distillation of cut tires (15–20 cm) in the old oil at 400 °C. Light and heavy oils can be obtained by this pyrolysis process. Operating costs are still very high [2].

This paper reviews the progress and challenges of the pyrolysis of plastic automotive waste along with future perspectives in comparison to thermal decomposition and pyrolysis. In addition, the factors affecting the pyrolysis process such as temperature, feedstock composition, ash content, calorific value were evaluated to improve the process of secondary usage of plastics from automobiles. Moreover, energetic recovery was also focused upon in order to make the pyrolysis process more economical and sustainable. The last form of both plastics and tire waste recycling is their incineration for energy recovery (quaternary recycling). In this process, the waste is incinerated and a certain amount of energy is recovered in the form of heat. This is generally the last possible process, when no more suitable application is obtainable. The incineration of plastics and tires also releases hazardous gases and creates toxic residues, which present a big environmental problem [12,22].

The aim of this work was to recover selected plastic materials and rubber from tires as waste from the automotive industry by using the tertiary (pyrolysis) and the quaternary (calorimetry) recovering process. The goal of this paper was to describe individual products of pyrolysis, too.

## 2. Materials and Methods

### 2.1. Material

#### 2.1.1. Rubber

Sample 1: Granulate from recycled tire (size from 1.0 to 3.0 mm)—sample 1
Sample 2: Granulate from recycled tire (size from 3.1 to 6.0 mm)—sample 2
The granulate from recycled tires was produced by AVE SK-Kechnec plant Slovakia.

#### 2.1.2. Plastics

Analyzed plastic waste materials were taken away from the vehicle scrap yard.
Plexiglass from the dashboard—sample 3
Perforated headlamp or reflector—sample 4
Inner fender—sample 5
Shielding part of the dashboard—sample 6
Front bumper-type 1—sample 7
Front bumper-type 2—sample 8
Car interior pillar panel—sample 9
Interior accessories—comfort equipment for seats-sample 10
Heating blower of cars—sample 11
Plastic wheel hub—sample 12

### 2.2. Methods

#### 2.2.1. Thermogravimetric Analysis

Thermogravimetric analysis (TG) took place in a nitrogen atmosphere (with the nitrogen purity of 3.0 with the flow rate of 50 mL/min^−1^) with the same method in the case of all samples. The weight of samples ranged between 38.170–41.620 mg. The measurement was carried out at the temperatures ranging between 30–800 °C in three segments. During the first 3 minutes and at the temperature of 30 °C—isothermal segment, the sample was stabilized. Subsequently, the thermodynamic segment continued at the heating speed of 10 °C/min. When the temperature of 800 °C was reached, the measurement was finished with the isothermal segment, and the final temperature of 800 °C was maintained for 3 min.

#### 2.2.2. Pyrolysis and GC-MS Analysis (Py-GC-MS)

The pyrolysis was performed by using the Pyroprobe 5150 Series (CDS Analytical Inc., Oxford, MA, USA). The pyrolyzer was interfaced (interface temperature of 150 °C). For the pyrolysis, approximately 1.2–2.1 mg of the sample were put in the quartz tube. The sample was closed with quartz wool on both sides. The pyrolysis temperature of the given samples was determined following the fastest material decomposition resulting from the thermogravimetric analysis. In these cases, the speed of heating was 10 °C/ms, final temperature was 500 °C and the retention time was 15 s.

The pyrolysis products were analyzed by the method GC-MS using the following devices: the gas chromatograph GC 7890A Agilent Technologies (Agilent, Santa Clara, CA, USA) and the mass spectrometer with ion source Agilent Technologies model 5975C (MSD) (Agilent, Santa Clara, CA, USA). The conditions of GC were: columns HP-5MS (30 m × 250 μm × 0.25 μm), carrier gas: helium (constant flow rate 2 mL·min^−1^), temperature program: from 60 °C (1 min) to 280 °C (16 °C·min^−1^) 280 °C (2 min), the temperature of the injector 280 °C in the split mode (70:1). The software ChemStation E 02.01.1177 (Agilent, Santa Clara, CA, USA) was used to record and evaluate the measured data. The components were identified comparing the measured mass spectra to the NIST and Wiley mass spectral libraries.

#### 2.2.3. Calorimetry

Waste plastics and granulates from tires were analyzed by using the calorimeter C 200 (IKA®-Werke GmbH & Co. KG, Staufen, Germany) and evaluated by the Cal Win software (IKA®-Werke GmbH & Co. KG, Staufen, Germany according to the standard STN ISO 1928 (44 1352) [26]. The percentage of ash content was calculated as the difference between the weight of the original sample before incineration and the residue after incineration in the calorimeter. Measurements were performed on four replicates per sample.

## 3. Results and Discussion

### 3.1. TG Analysis

The changes that occurred in polymeric materials with a gradual increase of the load temperature can be monitored by thermogravimetric analysis. Physico-chemical changes of the tested samples are associated with degradation reactions, formation of volatile degradation products and weight loss. One of the main objectives of TG analysis was to obtain information on suitable pyrolysis temperatures. Based on this, we chose the temperature reached at the fastest decay of the sample, obtained from the DTG curve.

**Figure 1:**
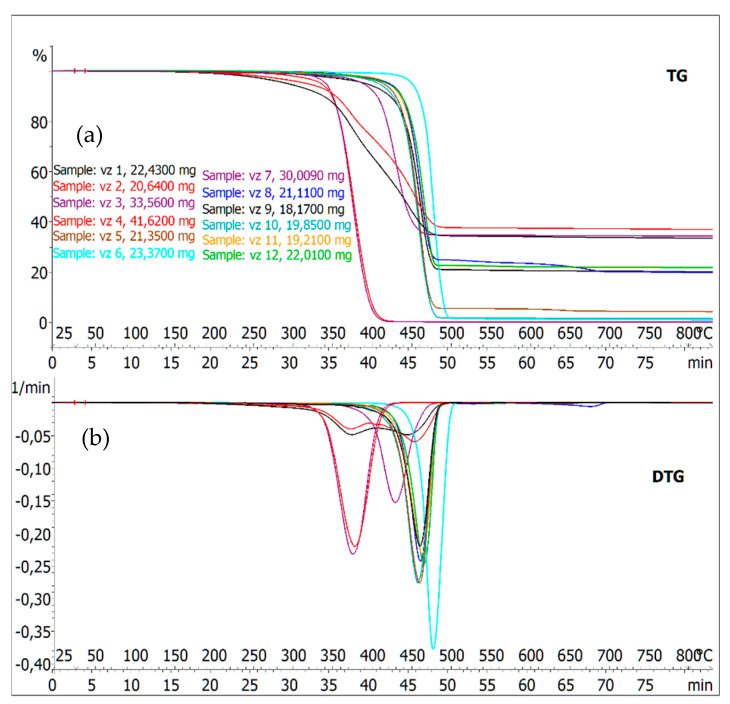
(**a**) Dynamic thermogravimetric curves (TG) of samples in an inert atmosphere, (**b**) derivation of thermogravimetric curves (DTG).

Two rubber samples (granulate from recycled tire) and ten different plastic materials originating from the automotive industry were analyzed by dynamic thermogravimetry. The TG and corresponding DTG curves of synthetic polymer samples obtained under the same heating rate conditions (10 °C·min^−1^) are shown in Figure 1.

Thermogravimetric analysis of the investigated materials provides typical decomposition curves of synthetic polymers. The slope of these curves characterizes the rate of weight changes. The first derivation of the TG curve represents the rate of weight loss of the sample as a function of temperature change.

In the thermolysis process, it was possible to observe only one degree of decomposition in the case of samples 3–12. In the case of samples 1 and 2, it was possible to observe the decomposition process via DTG curves in two degradation stages. Fazli and Rodrigue [27] analyzed rubber with the addition of recycled tires and mentioned a similar trend in DTG curves.

The relative weight loss after reaching the temperature of 800 °C in an inert atmosphere, as well as the weight loss reached at a temperature where the maximum rate of weight loss of the samples was evident, are summarized in Table 2. The highest temperature of 480.4 °C at the maximum rate of weight loss was reached in sample 6. The peak temperature was related to the chemical structure of the material. Longer chain or higher molecular weight materials will have a higher peak temperature [28].

An asimilar pattern of DTG curves was observed especially at samples 5, 8, 9, 10, 11, 12. The decay rate of these samples was in a narrow range from 433 to 441 °C. The lowest thermal resistivity had samples 3 and 4, while the highest decay rates were reached at the temperature of 378 and 379 °C. Compared to others, these samples had the highest relative weight loss without any charring yield at the temperature of 800 °C. It meant related or identical composition.

The TG/DTG curves of samples 1 and 2 showed the same trends indicating the same pyrolysis behavior due to similar chemical bonds in their molecular structures [29]. Thermal degradation of samples 1 and 2 begins at a lower temperature compared to other samples. The lower initial temperature of thermal degradation may be due to the presence of volatile substances in materials composed of recycled tires [30]. Analysis of our samples shows that the granulate from the recycled tire (samples 1 and 2), the plexiglass from the dashboard (sample 3) and the perforated headlamp (sample 4) decompose more easily to form oil products.

### 3.2. Py-GC-MS Analysis

Analytical pyrolysis of synthetic polymers obtained from various parts of the automobiles provides comprehensive information on a wide range of organic compounds released under thermal stress of these materials without access of oxygen (Tables S1–S12). Such information may be important for the planned energy recovery of automotive wastes, for finding the application of separating valuable compounds from secondary processing. The main compounds released during pyrolysis were gaseous hydrocarbons. The main reason is the degradation mechanisms of accidental cleavage of the polymer chain [31]. The reaction started with depolymerization at higher temperatures to form a gaseous fraction containing CO, CO_2_ and hydrocarbons [32]. CO_2_ a CO, which formed most of the gas composition, were generated by self-oxidation from the rest of the organic material. Samples 1, 2 consist of similar types of hydrocarbon gases such as styrene, methylstyrene and D-limonene. Samples 3, 4 showed the highest proportion of methylmethacrylate.

### 3.3. Calorimetry

Calorimetry is one of the methodological opportunities to utilize waste materials. For plastics and rubbers, this is the last possibility of their recovery. Based on the results reported in Table 3, it can be stated that the determined calorific values of selected plastic and rubber materials ranged from 26.261 (front bumper type 1) to 45.245 (front bumper type 2) MJ/kg.

The front bumper type 1 sample contained a large amount of ash (18.31%) and according to Geffertová, Geffert [33] results, increasing the ash content values means reducing the calorific value of materials. The difference between the two mentioned samples is related to the both front bumper composition and their manufacture. The lowest calorific value (from 26.261 to 26.521 MJ/kg) had samples: plexiglass from the dashboard (PMMA), performed headlamp (PMMA) and the above mentioned front bumper type 1; and the highest (more than 40 MJ/kg) samples: inner fender, front bumper type 2, heating blower of cars and plastic wheel hun. According to many researchers [34,35,36], the calorific value of plastics is around 40 MJ/kg, which is comparable to fuels. The high calorific value is due to the high content of carbone (70%-PP and 80%-PE) and hydrogen (9–12%-PP, PE), and low ash content [34,37,38]. Higher values are observed only for natural gas (48 MJ/kg) and heating oil (43 MJ/kg) [34]. There are differences between calorific value of many materials. For example, coal has this value about 28 MJ/kg [34]; paper 14–17 MJ/kg [33,34,38] and paper sludge 5.7–7.8 MJ/kg [33], because of very high inorganic content (42–52%); tetrapacks 17.7–22.3 MJ/kg depend on the presence of PE foil (22.3 MJ/kg with foil) [33]; rubber 22.2 MJ/kg [38]; leather 19 MJ/kg [37]; textile 17.5 KJ/kg [38] and wood from 16.5 to 23 MJ/kg depending on the lignin and extractive content of the material [38,39,40,41,42,43].

Some of the analyzed samples had only a small ash content (lower than 3%): dashboard plexiglass and perforated headlamp that were prepared from PMMA material, front bumper type 2 (PP), heating blower of cars, plastic wheel hub and inner fender. Granulate from recycled tire contained approximately 7.5% ash. The biggest amount (more than 18%) of ash had other samples used as an interior accessory, and the front bumper type 1, in which it was determined a high content of inorganic fillings. With the development of the automotive industry, the requirements regarding the quality of automotive plastic parts are becoming more and more stringent. Weld line, buckling deformation, uneven texture, color irregularity, etc. are manufacturing defects resulting from time in case of large injection molded parts as automotive bumpers, outboards and dashboards. The solution is to add further treatment processes as burnishing and paint spraying, which consume a lot of material resources [44]. In the automotive industry there is very important to use parts with lower weight, better design, higher moldability, better rigidity, etc. Enhancements of PP compounds have been achieved by compounding PP with fillers, as well as through higher stereoregularity, fluidity and rubberization [45].

Non-catalytic pyrolysis is a common method for the recycling of polyolefinic plastics, however, high temperature (573−1173 K) and long reaction time are generally required, and a broad range of products including alkanes and alkenes, gases and aromatics are formed [19,46,47,48,49].

These types of compounds should be separated with selective distillation technique or modified with catalyst for better separation. The resulting application of such processes can lead to the recovery of waste materials, the substitution of inputs in the production of new plastics or in the production of substances suitable as waxes and fuels. An example of such an application is to obtain valuable chemicals, liquid fuels and waxes in high yields [50].

The reaction temperature for plastics decomposing is high and a variety of products are formed such as linear and branched alkanes and alkenes, aromatics, and gases via formation of the carbenium ion from alkanes by the strong acidity of the acid catalysts. Considering the necessity of alkene hydrogenation for the production of valuable liquid chemicals such as diesels and gasolines and severe deactivation of the catalysts, two-step pyrolysis-hydroprocessing, which means non-catalytic pyrolysis of plastics and catalytic hydroprocessing of the pyrolyzed oils, was proposed [51,52,53,54].

## 4. Conclusions

Nowadays, the disposal of waste from the automotive industry represents a considerable problem. Waste plastics and tires are very difficult to decompose. In the past, they were stored all over the world. Currently, this type of waste is either used as an alternative fuel or transformed into new products in the construction industry. This paper deals with tertiary and quaternary recycling of these wastes. Based on the previous results, we can state that plastic and tire waste from the automotive industry represents the material with a great potential in terms of chemical and energy conversion.

Based on the results of TG analysis, the highest temperature (480.4 °C) at the maximum rate of weight loss was reached in the plastic sample-the shielding part of the dashboard. The plexiglass from the dashboard and the perforated headlamp or reflector had the lowest thermal resistance, with the highest disintegration rate reaching the temperature of 378 and 379 °C. Thermal degradation of the granulate from recycled tires starts at lower temperatures. The decomposition process was possible to observe in two degradation stages using DTG curves.

Regarding the Py-GC-MS analysis, various gaseous hydrocarbons were obtained during the pyrolysis process. The main pyrolysis products of samples from recycled tires were similar types of hydrocarbon gases, such as styrene, methylstyrene and D-limonene. Plexiglass from the dashboard and perforated headlamp (reflector) showed the highest proportion of methyl methacrylate. These types of compounds should be separated with selective distillation technique or modified with catalyst for better separation. Final application Various polyolefins such as LDPE, HDPE and PP can be converted to valuable chemicals in high yields, and also plastic wastes could be also transformed to give high yields of the valuable chemicals. These results can be improved by using catalysts with selectively dissociated the inner C–C bonds in polyolefins without isomerization or aromatization, which should enable a high yield of the target valuable chemicals. This type of catalyst system should contribute to not only the suppression of plastic wastes but also to the utilization of plastic wastes as raw materials for the production of chemicals.

From the point of view of the calorimetry analysis, the calorific values of selected plastic and rubber waste materials ranged from 26.26 to 45.25 MJ/kg. The front bumper type 1 contains a large amount of ash (18.31%) and it has the lower calorific value of 26.26 compared to the calorific value (45.25 MJ/kg) of the front bumper type 2. An ash content increase means a decrease in the calorific value of these types of samples. The lowest ash content had the following samples: plexiglass from the dashboard and perforated headlamp (reflector). These samples also had the very low calorific value of 26.5 MJ/kg. The calorific value of selected waste plastics depends on the ash content and the material of which they are composed.

## Figures and Tables

**Table 1:** Plastic materials used in the automotive sector.

Plastic Material	Properties	Utilization	Source
Polypropylene-PP Polyethylene-PE	Low price; good adaptability; good performance and easy to recycle;	Thin-walled moldings; fuel tanks and hoses	[6]
Expanded polypropylene-EPP	Excellent mechanical properties-flexibility, compressive strength; ability of high energy absorption; accomplish the strictest criteria in the field of shock protection; thermal and sound insulation	In the car exterior and interior; as part of bumpers; seats; luggage compartments; headrests; carpet fillings	[3]
Polyvinyl chloride-PVC	High tensile strength; toughness; fire resistance; chemical resistance PVC without plasticizers (novodur) is resistant to water, acids, alkalis, organic compounds, oxygen, water vapour; high hardness; abrasion resistance; mechanical strength; good electrical insulating properties; high gloss and clarity; self-extinguishing; glues bonding and welding; undersides coating of cars and textile underlying	Softened as a surface layer of artificial leather; foil; moldings; profiles; hoses Unsoftened in the production of loaded moldings; profiles and sheets	[3,4,7]
Polyurethane-PUR	Increased comfort; corrosion resistance; insulation; sound absorption	Production of precision thin-walled moldings; parallel coupling sleeves and coupling dusters; for insulation and sealing tapes; textile lamination; packaging; insulation materials in construction industry; adhesives and fibers	[8]
Acrylonitrile butadiene styrene-ABS	Hard, shiny surface; attractive appearance; enables galvanic plating	For the complex and stressed moldings; grilles; radiator covers; ventilation; headlight frames; steering wheel covers; rear-view mirror housings; wheel hub covers; dashboards; large bonnet parts; safety panel cover layer; roof panel cover layer, vacuum drawn plates; surface and interior body parts	[3,9,10]
Copolymer of styrene and acrylonitrile-SAN	The most chemically resistant from the polystyrene materials and lasts for a long time even at a temperature of 85 °C	Covers with a good resistance to low temperatures; for glass fiber reinforced products; projector covers; cars interior	[2]
Polymethylmethacrylate-PMMA	Clarity; colorlessness in large thicknesses (92% light transmission); resistance to weathering, water, diluted acids and hydroxides; heat resistance up to 80 °C; low surface hardness	Glazing caravans and vehicles	[3]
Polycarbonate-PC	Technically important plastic construction; high light transmission; high impact strength; good electrical insulating properties; high mechanical tensile strength; low water absorption; resistance to UV radiation; chemical and dimensional stability up to 140 °C	Highly resistant moldings	[7]
Polyamide-PA	High hardness; toughness; abrasion resistance; good electrical insulating properties	Part of stressed parts of the handles; window controls and sliding bushes in the form of construction plastics such as bearings; gears; coils; anticorrosive coatings of metals; electrical insulating layers; cords for tires; conveyor belts; carpet fibers; nets	[2]

**Table 2:** The temperature of the maximum weight loss (T_max_) and the relative weight loss.

Sample	(T_max_) [°C]	Weight Loss at T_max_ [%]	Weight Loss at 800 °C [%]
1	375.9/452.8	21.04/55.57	66.67
2	374.7/459.3	16.13/50.89	61.77
3	379.2	56.06	100
4	377.5	52.39	100
5	463.4	61.52	95.84
6	480.4	53.13	99.06
7	431.6	36.53	65.75
8	464.4	43.94	80.38
9	464.1	50.48	80.08
10	461.1	65.51	98.42
11	466.5	49.34	77.76
12	467.4	53.72	78.31

**Table 3:** Calorific values and ash content of wastes tires and plastics.

Sample/Property	Calorific Value (MJ/kg)	Ash Content (%)
1	36.441 ± 0.783	7.36 ± 2.40
2	37.051 ± 0.565	7.51 ± 3.19
3	26.521 ± 0.012	0.12 ± 0.06
4	26.478 ± 0.085	0.05 ± 0.01
5	44.558 ± 0.131	2.39 ± 0.10
6	34.784 ± 0.063	18.32 ± 0.16
7	26.261 ± 0.061	18.31 ± 0.23
8	45.245 ± 0.050	0.80 ± 0.01
9	35.795 ± 0.045	20.56 ± 0.04
10	35.921 ± 0.055	19.71 ± 0.01
11	45.130 ± 0.092	0.76 ± 0.25
12	40.493 ± 0.050	1.27 ± 0.13

## Data Availability

Data sharing not applicable.

## Supplementary Materials

The following are available online at https://www.mdpi.com/article/10.3390/polym13132210/s1. Table S1: Identified substances after pyrolysis (500 °C) of sample 1. Table S2: Identified substances after pyrolysis (500 °C) of sample 2, Table S3: Identified substances after pyrolysis (500 °C) of sample 3, Table S4: Identified substances after pyrolysis (500 °C) of sample 4, Table S5: Identified substances after pyrolysis (500 °C) of sample 5, Table S6: Identified substances after pyrolysis (500 °C) of sample 6, Table S7: Identified substances after pyrolysis (500 °C) of sample 7, Table S8: Identified substances after pyrolysis (500 °C) of sample 8, Table S9: Identified substances after pyrolysis (500 °C) of sample 9, Table S10: Identified substances after pyrolysis (500 °C) of sample 10, Table S11: Identified substances after pyrolysis (500 °C) of sample 11, Table S12: Identified substances after pyrolysis (500 °C) of sample 12.
