# Supplementary material for: Recycling of Wastes Plastics and Tires from Automotive Industry
Source: Polymers (Basel). 2021 Jul 3;13(13):2210. doi: 10.3390/polym13132210 (PMC8271936; doi:10.3390/polym13132210)
Supplement: Supplementary file 1 [file polymers-13-02210-s001.zip › polymers-1282248-supplementary.pdf]

Table S1-12. Identified substances.

Table S1. Identified substances after pyrolysis (500 °C) of sample 1.

RT (min)	Area (Ab*s)	Hit Name	Mol Weight (amu)	CAS Number
1.873	68263776	1,3-Butadiene	54.047	000106-99-0
1.946	185017493	Isoprene	68.063	000078-79-5
2.208	21460590	1,3-Pentadiene, 3-methyl-, (E)-	82.078	002787-43-1
2.266	54773257	Benzene	78.047	000071-43-2
2.607	26654831	Bicyclo[4.1.0]hept-2-ene	94.078	002566-57-6
2.707	107730535	Toluene	92.063	000108-88-3
3.031	10408376	4-Methyl-1,4-heptadiene	110.110	013857-55-1
3.100	65428323	Cyclohexene, 4-ethenyl-	108.094	000100-40-3
3.220	11878612	Tricyclo[3.2.1.0(1,5)]octane	108.094	019074-25-0
3.320	32465156	Ethylbenzene	106.078	000100-41-4
3.357	50307834	o-Xylene	106.078	000095-47-6
3.540	266438146	Styrene	104.063	000100-42-5
3.797	63594874	Benzene, (1-methylethyl)-	120.094	000098-82-8
3.949	6733814	7-Methylenebicyclo[4.2.0]octane	122.110	1000211-11-2
3.996	16198667	Benzene, 2-propenyl-	118.078	000300-57-2
4.075	32144657	Cyclohexene, 4-ethenyl-1,4-dimethyl-	136.125	001743-61-9
4.132	16615373	Benzene, 1,2,4-trimethyl-	120.094	000095-63-6
4.300	199762125	.alpha.-Methylstyrene	118.078	000098-83-9
4.473	9726871	3-Carene	136.125	013466-78-9
4.541	35713902	Bicyclo[4.2.0]oct-1-ene, 7-endo-ethenyl-	134.110	1000142-18-3
4.672	27221560	2,6-Dimethyl-1,3,5,7-octatetraene, E,E-	134.110	000460-01-5
4.725	138869403	D-Limonene	136.125	005989-27-5
4.809	9879346	Benzene, cyclopropyl-	118.078	000873-49-4
4.908	7739984	1-Propyne, 3-phenyl-	116.063	010147-11-2
4.971	11726505	Benzenepropanal	134.073	000104-53-0
5.270	19823854	Benzene, (2-methyl-1-propenyl)-	132.094	000768-49-0
5.485	14473863	1,3,8-p-Menthatriene	134.110	018368-95-1
5.600	12736984	Cyclohexane, 1,2,4-triethenyl-	162.141	002855-27-8
5.715	5598955	1,5,5-Trimethyl-6-methylene-cyclohexene	136.125	000514-95-4
5.757	4585248	Benzene, (2-methyl-2-propenyl)-	132.094	003290-53-7
5.810	5887439	Benzene, 4-pentenyl-	146.110	001075-74-7
5.873	17193083	2-Methyl-7-exo-vinylbicyclo[4.2.0]oct-1(2)-ene	148.125	107914-89-6
5.936	7001450	1,4,8-Dodecatriene, (E,E,E)-	162.141	024252-85-5
5.957	6364632	Cyclopropane, 1-butylidene-2-(2-butylcyclopropyl)-	192.188	091509-01-2
6.103	8719355	Cyclooctene, 5,6-diethenyl-, cis-	162.141	053264-72-5
6.177	3289764	Benzene, (1-methyl-1-butenyl)-	146.110	053172-84-2
6.213	9527401	Benzene, (2-cyclopropylethenyl)-	144.094	1000142-01-6
6.282	7453430	1H-Indene, 1,1-dimethyl-	144.094	018636-55-0
6.727	9686048	Benzene, cyclohexyl-	160.125	000827-52-1
6.785	6248205	8-Methylene-3-oxatricyclo[5.2.0.0(2,4)]nonane	136.089	1000211-14-3
7.167	3124065	Benzene, 1-cyclopenten-1-yl-	144.094	000825-54-7
7.351	2876013	Benzene, 1-hexynyl-	158.110	001129-65-3
7.461	3936689	Benzene, 3-cyclohexen-1-yl-	158.110	004994-16-5

Table S2. Identified substances after pyrolysis (500°C) of sample 2.

RT (min)	Area (Ab*s)	Hit Name	Mol Weight (amu)	CAS Number
1.863	85930369	1,3-Butadiene	54.047	000106-99-0
1.931	140838265	Isoprene	68.063	000078-79-5
2.203	15388189	(Z),(Z)-2,4-Hexadiene	82.078	006108-61-8
2.261	44870182	Benzene	78.047	000071-43-2
2.596	19539801	Cyclopentane, 1,3-bis(methylene)-	94.078	059219-48-6
2.707	110876274	Toluene	92.063	000108-88-3
2.906	9890016	9,12-Octadecadien-1-ol, (Z,Z)-	266.261	000506-43-4
3.089	74511434	Cyclohexene, 4-ethenyl-	108.094	000100-40-3
3.210	8056919	Tricyclo[3.2.1.0(1,5)]octane	108.094	019074-25-0
3.315	34681687	Benzene, 1,3-dimethyl-	106.078	000108-38-3
3.341	34863488	Ethylbenzene	106.078	000100-41-4
3.530	286432746	Styrene	104.063	000100-42-5
3.797	12096562	2-Nonen-4-yne, (Z)-	122.110	056392-46-2
3.991	11276725	Benzene, 2-propenyl-	118.078	000300-57-2
4.069	31724311	Cyclohexene, 1-methyl-4-(1-methylethenyl)-, (S)-	136.125	005989-54-8
4.295	28310930	.alpha.-Methylstyrene	118.078	000098-83-9
4.405	9498172	cis-2,6-Dimethyl-2,6-octadiene	138.141	002492-22-0
4.536	3637846	3-Methylenecycloheptene	108.094	034564-56-2
4.557	6399403	1,5-Hexadiene, 2,5-dimethyl-3-methylene-	122.110	059131-13-4
4.709	92495699	D-Limonene	136.125	005989-27-5
4.803	3690886	1-(2-Methylphenyl)ethanol	136.089	007287-82-3
4.966	3620518	Benzenepropanal	134.073	000104-53-0
5.265	5522385	Benzene, 1-methyl-4-(1-methylethenyl)-	132.094	001195-32-0
5.480	4000302	Cyclohexane, 1,2,4-triethenyl-	162.141	002855-27-8
5.600	4288470	1,2-Di(prop-2-ynyl)cyclohexane	160.125	220078-91-1
5.810	3824403	Benzene, 4-pentenyl-	146.110	001075-74-7
5.878	8066250	2-Methyl-7-exo-vinylbicyclo[4.2.0]oct-1(2)-ene	148.125	107914-89-6
5.930	1692792	10-Oxatricyclo[4.2.1.1(3,9)]dec-4-ene, 9-ethenyl-	162.104	138146-11-9
5.951	2048955	n.i.	n.i.	n.i.
6.098	3415552	Cyclooctene, 5,6-diethenyl-, cis-	162.141	053264-72-5
6.208	4499519	Benzene, 1-cyclopenten-1-yl-	144.094	000825-54-7
6.282	2513420	1H-Indene, 1-ethenyl-2,3-dihydro-	144.094	051783-46-1
6.418	2932538	Benzene, 1-(1-methylethenyl)-3-(1-methylethyl)-	160.125	001129-29-9
6.517	3377675	Benzene, cyclopentyl-	146.110	000700-88-9
6.727	4323310	Benzene, 4-hexenyl-	160.125	023086-43-3
6.785	2135737	6-[(1Z)-1,3-Butadienyl]-1,4-cycloheptadiene	146.110	084899-15-0
6.863	1824347	Benzene, cyclohexyl-	160.125	000827-52-1
7.351	2596680	Benzene, 1-hexynyl-	158.110	001129-65-3
7.461	5243797	Benzene, 3-cyclohexen-1-yl-	158.110	004994-16-5

Table S3. Identified substances after pyrolysis (500 °C) of sample 3.

RT (min)	Area (Ab*s)	Hit Name	Mol Weight (amu)	CAS Number
2.125	106718335	2-Propenoic acid, methyl ester	86.037	000096-33-3
2.392	1208244534	Methyl methacrylate	100.052	000080-62-6
2.618	2076021	2-Propenoic acid, 2-methyl-, 2-hydroxypropyl ester	144.079	000923-26-2

2.728	11928349	Butanoic acid, 2-methyl-, methyl ester	116.084	000868-57-5
2.827	4705501	3-Butenoic acid, 3-methyl-, methyl ester	114.068	025859-52-3
2.880	4729123	Butanoic acid, 2-methylene-, methyl ester	114.068	002177-67-5
3.881	7277340	4-Pentenoic acid, 2,4-dimethyl-, methyl ester	142.099	034998-29-3
4.295	7043519	2,2,3,3-Tetramethylcyclopropanecarboxylic acid, 4-methylcyclohexyl ester	238.193	1000221-97-5
4.772	7968212	6-Methyl-3,5-heptadiene-2-one	124.089	001604-28-0
4.814	7176857	Cyclopentanecarboxylic acid, 3-methyl-4-methylene-, methyl ester	154.099	062185-62-0
4.887	2981775	Cyclopentanecarboxylic acid, 3-methyl-4-methylene-, methyl ester, trans-	154.099	087753-70-6
5.003	5192455	Butanedioic acid, methyl-, dimethyl ester	160.074	001604-11-1
5.197	2418244	Cyclohexanecarboxylic acid, 1-ethyl-, methyl ester	170.131	004630-81-3
5.307	12776685	Butanedioic acid, methylene-, dimethyl ester	158.058	000617-52-7
5.574	2258107	Cyclooctane	112.125	000292-64-8
6.287	5665304	Pentanedioic acid, 2,4-dimethyl-, dimethyl ester	188.105	002121-68-8
6.371	27361225	1,2-Cyclopentanedicarboxylic acid, dimethyl ester, trans-(.+/-.)-	186.089	080656-12-8
6.560	2071417	2-Aminoimidazole-4-carboxylic acid, methyl ester	141.054	1000129-59-6
6.769	34203042	3-Furancarboxylic acid, 2-methyl-, methyl ester	140.047	006141-58-8
6.989	299851	2-Pentenedioic acid, dimethyl ester	158.058	005164-76-1
7.252	26283687	2-Pentenoic acid, 4-methyl-	114.068	010321-71-8
7.351	8805730	Hexanedioic acid, 2-methyl-5-methylene-, dimethyl ester	200.105	004513-62-6
7.466	6325673	2,3,4,5-Tetramethylcyclopent-2-en-1-ol	140.120	082061-20-9
7.519	12813660	2-Cyclopentene-1-carboxylic acid, 1-methyl-, methyl ester	140.084	068317-73-7
7.582	9054926	4,4-Dimethoxy-2,5-cyclohexadien-1-one	154.063	000935-50-2
7.697	4426045	Cyclopentanecarboxylic acid, 2-methyl-4-methylene-, methyl ester	154.099	074764-24-2
7.739	4841611	3-Cyclopentene-1,2-dicarboxylic acid, 4-methyl-, dimethyl ester, trans-	198.089	080137-87-7
7.886	4376414	3-Oxabicyclo[3.3.0]octan-2-one, 6-methylene-7-methyl-	152.084	1000155-60-1
8.913	1857879	1-Buten-1-ol, 2-methyl-4-(2,6,6-trimethyl-1-cyclohexen-1-yl)-, formate, (E)-	236.178	021730-91-6
10.517	2496541	Phenol, 2-(1,1-dimethylethyl)-4-methyl-	164.120	002409-55-4
11.099	17955641	3-Ethoxybenzhydrazide	180.090	027830-16-6

Table S4. Identified substances after pyrolysis (500 °C) of sample 4.

RT (min)	Area (Ab*s)	Hit Name	Mol Weight (amu)	CAS Number
2.141	10317856	Methyl propionate	88.052	000554-12-1
2.387	1100051789	Methyl methacrylate	100.052	000080-62-6
2.717	9138280	Butanoic acid, 2-methyl-, methyl ester	116.084	000868-57-5
2.817	2916135	3-Butenoic acid, 3-methyl-, methyl ester	114.068	025859-52-3
2.869	3117347	2-Butenoic acid, 2-methyl-, methyl ester, (Z)-	114.068	005953-76-4
3.876	5114053	4-Pentenoic acid, 2,4-dimethyl-, methyl ester	142.099	034998-29-3
4.290	5840215	2,2,3,3-Tetramethylcyclopropanecarboxylic acid, 4-methylcyclohexyl ester	238.193	1000221-97-5

4.809	3878123	Cyclopentanecarboxylic acid, 3-methyl-4-methylene-, methyl ester, cis-	154.099	087753-69-3
5.008	3539346	Butanedioic acid, methyl-, dimethyl ester	160.074	001604-11-1
5.191	2117503	3-Isopropyl-5-methyl-hex-4-en-2-one	154.136	077142-85-9
5.307	7265531	Butanedioic acid, methylene-, dimethyl ester	158.058	000617-52-7
6.051	1365757	Pentanedioic acid, 2,4-dimethyl-, dimethyl ester	188.105	002121-68-8
6.124	5590825	1-Dodecene	168.188	000112-41-4
6.434	2672353	2,4-Octadienoic acid, 7-hydroxy-, methyl ester, [R-(E,E)]-	170.094	069734-24-3
6.947	5504482	Dimethyl ethylidenemalonate	158.058	017041-60-0
7.251	16236166	Cyclopropanecarboxylic acid, methyl ester	100.052	002868-37-3
7.304	2478301	2-Pentenoic acid, 4-methyl-	114.068	010321-71-8
7.356	5159486	Hexanedioic acid, 2-methyl-5-methylene-, dimethyl ester	200.105	004513-62-6
7.414	20372248	5-Methyl-2-ethenyl-cyclohexane-1-carboxylic acid	168.115	1000144-53-6
7.582	5581136	4,4-Dimethoxy-2,5-cyclohexadien-1-one	154.063	000935-50-2
7.697	2832866	2-Propanone, 1-(1-cyclohexen-1-yl)-3-ethoxy-	182.131	051149-72-5
8.012	3997775	Cyclopentanecarboxylic acid, 2-methyl-4-methylene-, methyl ester	154.099	074764-24-2
10.847	4375681	1H-Perimidine-1-ethanol	212.095	020957-13-5
11.099	13665983	2-Naphthalenemethanol, 2,3,4,4a,5,6,7,8-octahydro-.alpha.,.alpha.,4a,8-tetramethyl-, [2R-(2.alpha.,4a.beta.,8.beta.)]-	222.198	063891-61-2

Table S5. Identified substances after pyrolysis (500°C) of sample 5.

RT (min)	Area (Ab*s)	Hit Name	Mol Weight (amu)	CAS Number
1.989	65932388	1-Butanol, 2-methyl-	88.089	000137-32-6
2.114	132870613	1-Pentene, 2-methyl-	84.094	000763-29-1
2.272	56179810	(Z),(Z)-2,4-Hexadiene	82.078	006108-61-8
2.382	40937528	1-Heptene	98.110	000592-76-7
2.675	47220106	1-Heptene, 4-methyl-	112.125	013151-05-8
2.733	10123869	Heptane, 4-methyl-	114.141	000589-53-7
2.796	42621680	2,4-Dimethyl-1-hexene	112.125	016746-87-5
2.979	22239610	2,2-Dimethyl-3-heptene trans	126.141	019550-75-5
3.168	466355602	2,4-Dimethylhept-1-ene	126.141	1000411-46-2
3.299	25491289	Cyclohexane, 1,3,5-trimethyl-, (1.alpha.,3.alpha.,5.beta.)-	126.141	001795-26-2
3.404	17715256	Cyclohexene, 3,3,5-trimethyl-	124.125	000503-45-7
3.530	23122796	Ethyl 2-ethylbutyl carbonate	174.126	1000373-79-7
3.545	30985435	1-Dodecanone, 1-cyclopropyl-	224.214	019873-44-0
3.708	28985377	2-Cyclohexen-1-one, 4,5-dimethyl-	124.089	005715-25-3
4.017	6542540	Cyclodecane	140.157	000293-96-9
4.075	4969275	Nonane, 4-methyl-	142.172	017301-94-9
4.285	5447267	2-Methyl-1-nonene	140.157	002980-71-4
4.337	11151677	1-Decene	140.157	000872-05-9
4.368	15063904	2-Decene, 4-methyl-, (Z)-	154.172	074630-30-1
4.400	16646756	1,1,4-Trimethylcyclohexane	126.141	007094-27-1
4.494	6655241	Octane, 3,3-dimethyl-	142.172	004110-44-5
4.531	6802722	Heptane, 5-ethyl-2-methyl-	142.172	013475-78-0

4.725	4073142	4-Decene, 5-methyl-, (E)-	154.172	062338-51-6
4.804	3800513	Cyclohexane, 1,1-dimethyl-2-propyl-	154.172	081983-71-3
4.856	5322938	Cyclopentane, 1,1,2-trimethyl-	112.125	004259-00-1
4.950	7841361	Cyclopentane, 1-ethyl-1-methyl-	112.125	016747-50-5
5.081	3100020	2,6-Octadienal, 3,7-dimethyl-, (Z)-	152.120	000106-26-3
5.139	51405343	Cyclohexane, 1,1,3,5-tetramethyl-, trans-	140.157	050876-31-8
5.176	26420407	5-Ethyl-1-nonene	154.172	019780-74-6
5.233	9224671	Cyclopropane, 1-methyl-2-pentyl-	126.141	041977-37-1
5.302	4381628	Undecane	156.188	001120-21-4
5.569	13702974	Cyclohexane, 1,2-diethyl-1-methyl-	154.172	061141-79-5
5.627	4327844	Cyclopentane, 1,1,3,4-tetramethyl-, trans-	126.141	020309-77-7
5.731	2594322	6-Dodecene, (E)-	168.188	007206-17-9
5.789	23204205	1,7-Nonadiene, 4,8-dimethyl-	152.157	062108-28-5
5.931	2459770	1-Isopropyl-1,4,5-trimethylcyclohexane	168.188	219783-06-9
6.072	4971480	3-Undecene, 10-methyl-	168.188	1000061-84-5
6.130	5558837	Cyclododecane	168.188	000294-62-2
6.203	3361726	Dodecane	170.203	000112-40-3
6.355	4230066	n.i.	n.i.	n.i.
6.413	2365377	n.i.	n.i.	n.i.
6.491	2514145	Cyclopentane, 1-pentyl-2-propyl-	182.203	062199-51-3
6.523	4585596	Cyclopropane, 1-heptyl-2-methyl-	154.172	074663-91-5
6.596	3081553	1-Decanol, 2-methyl-	172.183	018675-24-6
6.733	1984915	Cyclohexane, 1,1,2,3-tetramethyl-	140.157	006783-92-2
6.801	1897059	2-Butene, 2,3-dimethyl-	84.094	000563-79-1
6.963	5244926	4-Isopropylcyclohexanone	140.120	005432-85-9
7.000	2501732	1-Tridecene	182.203	002437-56-1
7.147	55358557	2,3-Dimethyl-3-heptene, (Z)-	126.141	059643-73-1
7.210	10125148	n.i.	n.i.	n.i.
7.288	34838028	4-Isopropyl-1,3-cyclohexanedione	154.099	062831-62-3
7.508	5975896	3-Decene, 2,2-dimethyl-, (E)-	168.188	055499-02-0
7.697	1590013	3-Hexene, 2,2,5,5-tetramethyl-, (Z)-	140.157	000692-47-7
7.739	7145426	Cyclopropanemethanol, 2-methyl-2-(4-methyl-3-pen-tenyl)-	168.151	098678-70-7
7.839	1928421	2-Tetradecene, (E)-	196.219	035953-54-9
7.881	2597807	Cyclooctane, 1,4-dimethyl-, cis-	140.157	013151-99-0
8.179	1847858	Cyclohexane, 1-ethyl-2-propyl-	154.172	062238-33-9
8.641	1248655	Cyclopentadecane	210.235	000295-48-7
8.887	5755420	Cyclohexane, 1,2,4-trimethyl-	126.141	002234-75-5
9.091	1903747	n.i.	n.i.	n.i.
9.437	2784218	n.i.	n.i.	n.i.

Table S6. Identified substances after pyrolysis (500°C) of sample 6.

RT (min)	Area (Ab*s)	Hit Name	Mol Weight (amu)	CAS Number
1.994	15444946	1-Butanol, 2-methyl-	88.089	000137-32-6
2.057	13360762	n.i.	n.i.	n.i.
2.141	34211078	1-Pentene, 2-methyl-	84.094	000763-29-1
2.282	11150566	1H-Pyrrole, 1-methyl-	81.058	000096-54-8
2.696	11799703	Heptane, 4-methyl-	114.141	000589-53-7

2.985	3472211	1-Hexene, 3,3,5-trimethyl-	126.141	013427-43-5
3.179	198103392	2,4-Dimethyl-1-heptene	126.141	019549-87-2
3.304	5952450	Cyclohexane, 1,3,5-trimethyl-, (1.alpha.,3.alpha.,5.beta.)-	126.141	001795-26-2
3.545	11836398	2-Pentene, 3-methyl-, (Z)-	84.094	000922-62-3
3.713	3922574	Pyruvic acid, 3-hexenyl ester	170.094	1000132-46-3
4.368	5515814	1,1,4-Trimethylcyclohexane	126.141	007094-27-1
4.395	5260216	1-Hexene, 3,3-dimethyl-	112.125	003404-77-1
4.494	2617017	Octane, 3,5-dimethyl-	142.172	015869-93-9
4.531	2516226	Decane, 3,3,8-trimethyl-	184.219	062338-16-3
5.139	22142183	Cyclohexane, 1,1,3,5-tetramethyl-, cis-	140.157	050876-32-9
5.176	12040496	3-Heptene, 4-methyl-	112.125	004485-16-9
5.569	4862106	2,3-Dimethyl-3-heptene, (Z)-	126.141	059643-73-1
5.627	1586774	2,3-Dimethyl-3-heptene	126.141	1000113-49-3
5.789	2766651	1,7-Nonadiene, 4,8-dimethyl-	152.157	062108-28-5
6.360	1639905	2-Undecene, 4-methyl-	168.188	091695-32-8
6.963	1428035	4-Isopropylcyclohexanone	140.120	005432-85-9
7.141	37479312	n.i.	n.i.	n.i.
7.283	34123971	Cyclohexane, 1,1-dimethyl-2-propyl-	154.172	081983-71-3
7.514	3437142	3-Decene, 2,2-dimethyl-, (E)-	168.188	055499-02-0
7.739	3268248	Cyclopropanemethanol, 2-methyl-2-(4-methyl-3-pentenyl)-	168.151	098678-70-7
8.892	8034954	Cyclohexane, 1,2,4-trimethyl-	126.141	002234-75-5
9.437	1926570	1,6-Octadiene, 2,5-dimethyl-, (E)-	138.141	068702-25-0
10.439	2045113	n.i.	n.i.	n.i.
10.627	1430770	Cyclopentane, hexyl-	154.172	004457-00-5

Table S7. Identified substances after pyrolysis (500°C) of sample 7.

RT (min)	Area (Ab*s)	Hit Name	Mol Weight (amu)	CAS Number
1.983	40548090	1-Butanol, 2-methyl-	88.089	000137-32-6
2.114	92886937	1-Pentene, 2-methyl-	84.094	000763-29-1
2.272	28771247	(Z),(Z)-2,4-Hexadiene	82.078	006108-61-8
2.387	9677424	1-Heptene	98.110	000592-76-7
2.413	9077888	3-Ethyl-1,3-dimethyldiaziridine (trans)	100.100	1000283-16-1
2.680	20901436	Hexane, 2,3-dimethyl-	114.141	000584-94-1
2.733	4499109	Heptane, 4-methyl-	114.141	000589-53-7
2.817	25740536	2-Octene, (Z)-	112.125	007642-04-8
2.979	9987251	1-Hexene, 3,3,5-trimethyl-	126.141	013427-43-5
3.158	292046896	2,4-Dimethylhept-1-ene	126.141	1000411-46-2
3.299	13236185	Cyclohexane, 1,2,4-trimethyl-, (1.alpha.,2.beta.,4.beta.)-	126.141	007667-60-9
3.467	5802667	1-Heptene, 2,6-dimethyl-	126.141	003074-78-0
3.545	36287796	Styrene	104.063	000100-42-5
3.708	11090170	Pyruvic acid, 3-hexenyl ester	170.094	1000132-46-3
3.870	9660800	2-Propenoic acid, 2-methyl-, 2-methylpropyl ester	142.099	000097-86-9
4.017	4203105	Camphene	136.125	000079-92-5
4.290	4125765	Cyclobutanone, 2,3,3-trimethyl-	112.089	028290-01-9
4.337	6290163	1-Decene	140.157	000872-05-9
4.368	9252673	2-Undecene, 4-methyl-	168.188	091695-32-8

4.395	10205997	1,1,4-Trimethylcyclohexane	126.141	007094-27-1
4.531	6918788	Decane, 4-methyl-	156.188	002847-72-5
4.945	3607918	Bicyclo[3.1.1]heptan-3-one, 2,6,6-trimethyl-, (1.alpha.,2.beta.,5.alpha.)-	152.120	015358-88-0
5.081	2736795	6,11-Dimethyl-2,6,10-dodecatrien-1-ol	208.183	1000196-53-3
5.134	30316886	Cyclohexane, 1,1,3,5-tetramethyl-, cis-	140.157	050876-32-9
5.170	14604160	n.i.	n.i.	n.i.
5.233	5511816	1-Undecene	154.172	000821-95-4
5.301	2901588	Undecane	156.188	001120-21-4
5.569	8247877	Cyclohexane, 1,1,3,5-tetramethyl-, trans-	140.157	050876-31-8
5.626	2550262	2,3-Dimethyl-3-heptene, (Z)-	126.141	059643-73-1
5.789	12127126	1,7-Nonadiene, 4,8-dimethyl-	152.157	062108-28-5
5.925	2259054	4-Undecene, 2-methyl-, (E)-	168.188	028665-57-8
6.077	2214242	5-Undecene, 9-methyl-, (Z)-	168.188	074630-65-2
6.130	4940982	Cyclopropane, 1-methyl-2-octyl-	168.188	037617-26-8
6.203	1893933	Dodecane	170.203	000112-40-3
6.355	2409474	Cyclohexane, 2-ethyl-1,3-dimethyl-	140.157	007045-67-2
6.413	1403439	n.i.	n.i.	n.i.
6.491	953143	3-Tridecene, (Z)-	182.203	041446-53-1
6.523	1352375	Tridecane, 4-methyl-	198.235	026730-12-1
6.963	2975238	2,6-Octadienal, 3,7-dimethyl-, (Z)-	152.120	000106-26-3
7.000	1823235	1-Tridecene	182.203	002437-56-1
7.141	35793307	n.i.	n.i.	n.i.
7.210	6000168	4-Isopropyl-1,3-cyclohexanedione	154.099	062831-62-3
7.283	21892084	4-Decene, 3-methyl-, (E)-	154.172	062338-47-0
7.508	4033981	3-Decene, 2,2-dimethyl-, (E)-	168.188	055499-02-0
7.739	4447710	Cyclopropanemethanol, 2-methyl-2-(4-methyl-3-pentenyl)-	168.151	098678-70-7
8.892	4674981	n.i.	n.i.	n.i.
9.437	1819277	1,6-Octadiene, 2,5-dimethyl-, (E)-	138.141	068702-25-0

Table S8. Identified substances after pyrolysis (500 °C) of sample 8.

RT (min)	Area (Ab*s)	Hit Name	Mol Weight (amu)	CAS Number
1.994	31926662	1-Propene, 2-methyl-3-(1-methylethoxy)-	114.104	044744-50-5
2.057	18744694	2-Pentene, 4-methyl-	84.094	004461-48-7
2.140	44756411	1-Pentene, 2-methyl-	84.094	000763-29-1
2.277	23529152	n.i.	n.i.	n.i.
2.397	44293825	Methyl methacrylate	100.052	000080-62-6
2.680	19670861	Hexane, 2,3-dimethyl-	114.141	000584-94-1
2.738	4458709	Heptane, 4-methyl-	114.141	000589-53-7
2.817	22909762	2-Heptene, 5-methyl-	112.125	022487-87-2
2.979	7232580	2,2-Dimethyl-3-heptene trans	126.141	019550-75-5
3.173	190675910	2,4-Dimethyl-1-heptene	126.141	019549-87-2
3.304	8015296	Cyclohexane, 1,2,4-trimethyl-, (1.alpha.,2.beta.,4.beta.)-	126.141	007667-60-9
3.535	76812576	Styrene	104.063	000100-42-5
3.708	4518081	cis-3-Hexenyllactate	172.110	061931-81-5
3.750	2563789	n.i.	n.i.	n.i.
4.012	3913022	5-Eicosene, (E)-	280.313	074685-30-6

4.227	34473757	n-Butyl methacrylate	142.099	000097-88-1
4.311	11567033	.alpha.-Methylstyrene	118.078	000098-83-9
4.368	11164667	2-Decene, 4-methyl-, (Z)-	154.172	074630-30-1
4.494	2337396	Heptane, 3,3,5-trimethyl-	142.172	007154-80-5
4.531	2328785	Dodecane, 4,6-dimethyl-	198.235	061141-72-8
4.725	2003522	4-Decene, 5-methyl-, (E)-	154.172	062338-51-6
4.809	1741369	4-Decene, 2-methyl-, (Z)-	154.172	055499-07-5
4.976	5943015	n.i.	n.i.	n.i.
5.134	18115353	Cyclopentane, propyl-	112.125	002040-96-2
5.170	10602346	6-Dodecene, (E)-	168.188	007206-17-9
5.233	5310351	3-Undecene, (E)-	154.172	001002-68-2
5.569	4261246	Cyclohexane, 1,1,3,5-tetramethyl-, trans-	140.157	050876-31-8
5.789	6480543	2-Propen-1-one, 1-(2,2-dimethylcyclopropyl)-	124.089	077846-92-5
6.077	1734181	5-Undecene, 2-methyl-, (Z)-	168.188	074630-63-0
6.491	1836547	n.i.	n.i.	n.i.
6.523	1931915	2-Undecene, (Z)-	154.172	000821-96-5
6.963	1631212	n.i.	n.i.	n.i.
7.136	13811618	2,3-Dimethyl-3-heptene, (Z)-	126.141	059643-73-1
7.204	2585809	Hexane, 2,3,4-trimethyl-	128.157	000921-47-1
7.283	8753582	4-Isopropyl-1,3-cyclohexanedione	154.099	062831-62-3

Table S9. Identified substances after pyrolysis (500 °C) of sample 9.

RT (min)	Area (Ab*s)	Hit Name	Mol Weight (amu)	CAS Number
1.983	85467962	1-Butanol, 2-methyl-	88.089	000137-32-6
2.109	157073627	1-Pentene, 2-methyl-	84.094	000763-29-1
2.261	77757783	(Z),(Z)-2,4-Hexadiene	82.078	006108-61-8
2.413	45768668	2,4-Hexadiene, 2-methyl-	96.094	028823-41-8
2.665	57620291	Hexane, 2,3-dimethyl-	114.141	000584-94-1
2.733	11683460	1,4-Hexadiene, 4-methyl-	96.094	001116-90-1
2.780	30578829	1-Heptene, 2-methyl-	112.125	015870-10-7
2.958	33045718	2,2-Dimethyl-3-heptene trans	126.141	019550-75-5
3.168	580390756	2,4-Dimethyl-1-heptene	126.141	019549-87-2
3.283	31413648	Cyclohexane, 1,3,5-trimethyl-, (1.alpha.,3.alpha.,5.beta.)-	126.141	001795-26-2
3.393	10705583	2,4-Heptadiene, 2,4-dimethyl-	124.125	074421-05-9
3.456	12457795	Cyclohexene, 3,3,5-trimethyl-	124.125	000503-45-7
3.524	47933422	2-Pentene, 3-methyl-, (Z)-	84.094	000922-62-3
3.614	8149237	Bicyclo[3.1.1]heptan-2-one, 6,6-dimethyl-, (1R)-	138.104	038651-65-9
3.697	23838494	Pyruvic acid, 3-hexenyl ester	170.094	1000132-46-3
3.954	3617685	Cyclobutane, butyl-	112.125	013152-44-8
4.017	6481929	cis-3-Decene	140.157	019398-86-8
4.279	4253017	2-Methyl-1-nonene	140.157	002980-71-4
4.363	44439992	2-Decene, 4-methyl-, (Z)-	154.172	074630-30-1
4.489	7586297	Hexane, 3,3-dimethyl-	114.141	000563-16-6
4.526	7517474	Heptane, 5-ethyl-2-methyl-	142.172	013475-78-0
4.720	3462266	4-Undecene, (Z)-	154.172	000821-98-7
4.756	2440328	2-Undecene, 4-methyl-	168.188	091695-32-8
4.798	3619693	1-Ethyl-2,2,6-trimethylcyclohexane	154.172	071186-27-1

4.851	5483541	1-Undecene, 7-methyl-	168.188	074630-42-5
4.950	10577725	Cyclohexane, hexyl-	168.188	004292-75-5
4.997	3848291	Bicyclo[3.1.1]heptan-3-one, 2,6,6-trimethyl-, (1.alpha.,2.beta.,5.alpha.)-	152.120	015358-88-0
5.081	4033861	6-Octenoic acid, 3,7-dimethyl-, 3,7-dimethyl-6-octenyl ester	308.272	082766-40-3
5.139	64049666	n.i.	n.i.	n.i.
5.176	30803712	4-Dodecene	168.188	002030-84-4
5.228	5980826	Cyclopropane, 1-methyl-2-pentyl-	126.141	041977-37-1
5.301	2657828	Undecane	156.188	001120-21-4
5.537	4168161	Cyclopentane, 1-ethyl-1-methyl-	112.125	016747-50-5
5.569	22096954	Cyclohexane, 1,1,3,5-tetramethyl-, trans-	140.157	050876-31-8
5.653	2523471	4-Dodecene, (E)-	168.188	007206-15-7
5.726	2954759	Cyclooctane, 1,2-dimethyl-	140.157	013151-94-5
5.789	24330573	1,7-Nonadiene, 4,8-dimethyl-	152.157	062108-28-5
5.810	4952639	4-Dodecanol	186.198	010203-32-4
5.925	2905767	Cyclohexanecarboxylic acid, 4-propyl-, 4-cyanophenyl ester, trans-	271.157	062439-33-2
6.072	3647569	5-Undecene, 2-methyl-, (Z)-	168.188	074630-63-0
6.124	3949228	Cyclododecane	168.188	000294-62-2
6.355	8358366	1-Hexene, 3,3-dimethyl-	112.125	003404-77-1
6.449	2394812	4,8-Decadienal, 5,9-dimethyl-	180.151	000762-26-5
6.491	2198753	Carbonic acid, decyl tridecyl ester	384.360	1000383-16-2
6.523	5160007	Cyclohexane, 2-butyl-1,1,3-trimethyl-	182.203	054676-39-0
6.591	2843592	Carbonic acid, octadecyl prop-1-en-2-yl ester	354.313	1000383-11-5
6.733	2438106	Cyclopentane, 1-butyl-2-propyl-	168.188	062199-50-2
6.801	1311728	1-Octene, 3,7-dimethyl-	140.157	004984-01-4
6.885	3729895	(E)-4-Oxohex-2-enal	112.052	1000374-04-2
6.963	5934153	4-Hexen-1-ol, 5-methyl-2-(1-methylethenyl)-, (R)-	154.136	000498-16-8
7.031	2144282	2-Dodecene, 2-methyl-	182.203	055103-82-7
7.094	2377160	Cyclohexane, propyl-	126.141	001678-92-8
7.147	69007014	2,3-Dimethyl-3-heptene, (Z)-	126.141	059643-73-1
7.210	13027762	2-Acetylcyclopentanone	126.068	001670-46-8
7.288	43369262	4-Isopropyl-1,3-cyclohexanedione	154.099	062831-62-3
7.477	1947993	Cyclobutanecarboxylic acid, undec-2-enyl ester	252.209	1000299-13-6
7.508	7659468	3-Decene, 2,2-dimethyl-, (E)-	168.188	055499-02-0
7.697	2099849	Cyclohexane, 3-ethyl-5-methyl-1-propyl-	168.188	1000151-39-5
7.739	8945912	n.i.	n.i.	n.i.
7.881	1953495	11-Dodecen-1-ol, 2,4,6-trimethyl-, (R,R,R)-	226.230	027829-54-5
8.179	2068521	Cyclopropane, 1,1-dimethyl-2-nonyl-	196.219	041977-38-2
8.892	6329159	Cyclohexane, 1,1,2-trimethyl-	126.141	007094-26-0
9.097	2124044	n.i.	n.i.	n.i.

Table S10. Identified substances after pyrolysis (500°C) of sample 10.

RT (min)	Area (Ab*s)	Hit Name	Mol Weight (amu)	CAS Number
1.988	29015074	1-Butanol, 2-methyl-	88.089	000137-32-6
2.062	20266048	1-Pentene, 3-methyl-	84.094	000760-20-3
2.125	56338912	1-Pentene, 2-methyl-	84.094	000763-29-1

2.271	23880907	(Z),(Z)-2,4-Hexadiene	82.078	006108-61-8
2.675	22226200	Hexane, 2,3-dimethyl-	114.141	000584-94-1
2.738	5969997	Heptane, 4-methyl-	114.141	000589-53-7
2.780	10104374	2-Heptene, 6-methyl-	112.125	073548-72-8
2.979	12068419	2,2-Dimethyl-3-heptene trans	126.141	019550-75-5
3.163	350342700	2,4-Dimethylhept-1-ene	126.141	1000411-46-2
3.294	14547545	Cyclohexane, 1,3,5-trimethyl-, (1.alpha.,3.alpha.,5.beta.)-	126.141	001795-26-2
3.398	4088645	1,2,4,4-Tetramethylcyclopentene	124.125	065378-76-9
3.467	4755322	Cyclohexene, 3,3,5-trimethyl-	124.125	000503-45-7
3.535	23337867	2-Pentene, 3-methyl-, (Z)-	84.094	000922-62-3
3.703	10327325	3-Hexen-1-ol, acetate, (E)-	142.099	003681-82-1
4.368	21554658	2-Decene, 4-methyl-, (Z)-	154.172	074630-30-1
4.489	4655153	Decane, 2,8,8-trimethyl-	184.219	1000060-81-2
4.526	4498845	Octane, 3,3-dimethyl-	142.172	004110-44-5
4.725	1443034	4-Decene, 5-methyl-, (E)-	154.172	062338-51-6
4.803	1387883	Nonane, 2-methyl-3-methylene-	154.172	055499-08-6
4.950	4344887	1,7-Octadiene, 2,3,3-trimethyl-	152.157	1000150-47-7
4.997	1676624	Bicyclo[3.1.1]heptan-3-one, 2,6,6-trimethyl-, (1.alpha.,2.alpha.,5.alpha.)-	152.120	000547-60-4
5.081	1871306	4-Nonene, 2,3,3-trimethyl-, (Z)-	168.188	063830-68-2
5.139	37194761	n.i.	n.i.	n.i.
5.170	18808937	n.i.	n.i.	n.i.
5.228	2440402	Cyclopropane, 1-heptyl-2-methyl-	154.172	074663-91-5
5.537	1339010	1-Cyclohexylheptene	180.188	114614-83-4
5.569	9353697	2,3-Dimethyl-3-heptene, (Z)-	126.141	059643-73-1
5.626	2784599	Cyclohexane, 1,1,3,5-tetramethyl-, trans-	140.157	050876-31-8
5.789	14623878	1,7-Nonadiene, 4,8-dimethyl-	152.157	062108-28-5
5.925	1553261	n.i.	n.i.	n.i.
6.072	1344106	4-Undecene, 2-methyl-, (E)-	168.188	028665-57-8
6.292	605850	Cyclohexane, 2,4-diethyl-1-methyl-	154.172	061142-70-9
6.355	4754799	1-Hexene, 3,3-dimethyl-	112.125	003404-77-1
6.491	1185188	Cyclohexane, 1,2,4-trimethyl-	126.141	002234-75-5
6.523	2677980	Decane, 2-methyl-	156.188	006975-98-0
6.596	1314949	Nonane, 3-methyl-5-propyl-	184.219	031081-18-2
6.732	1288192	Cyclooctane, 1-methyl-3-propyl-	168.188	255885-37-1
6.884	1522520	Cyclohexanecarbonyl chloride	146.050	002719-27-9
6.963	2170345	Cyclopropanecarboxaldehyde, 2-methyl-2-(4-methyl-3-pentenyl)-, trans-(.+-.)-	166.136	097231-35-1
7.031	1297750	1-Cyclohexyl-2-methyl-prop-2-en-1-one	152.120	025183-82-8
7.141	43866543	n.i.	n.i.	n.i.
7.209	9167398	n.i.	n.i.	n.i.
7.283	28594049	4-Isopropyl-1,3-cyclohexanedione	154.099	062831-62-3
7.508	4524819	3-Decene, 2,2-dimethyl-, (E)-	168.188	055499-02-0
7.739	5888500	Cyclohexane, 1,1,3,5-tetramethyl-, cis-	140.157	050876-32-9
8.184	1097946	Cyclopentane, 1-butyl-2-propyl-	168.188	062199-50-2
8.892	4361830	n.i.	n.i.	n.i.
9.097	1523164	1-Undecene, 7-methyl-	168.188	074630-42-5
9.442	1537635	1,6-Octadiene, 2,5-dimethyl-, (E)-	138.141	068702-25-0
10.438	1343690	Cyclooctane, butyl-	168.188	016538-93-5

Table S11. Identified substances after pyrolysis (500 °C) of sample 11.

RT (min)	Area (Ab*s)	Hit Name	Mol Weight (amu)	CAS Number
1.994	5530753	1,3-Butadiene	54.047	000106-99-0
2.046	3365582	2-Pentene, (E)-	70.078	000646-04-8
2.172	17705640	1-Hexene	84.094	000592-41-6
2.418	15454227	1-Heptene	98.110	000592-76-7
2.549	2293026	Cyclohexane, methyl-	98.110	000108-87-2
2.859	6426957	1-Octene	112.125	000111-66-0
2.906	5512039	Octane	114.141	000111-65-9
3.456	1295243	1,8-Nonadiene	124.125	004900-30-5
3.519	6146285	1-Nonene	126.141	000124-11-8
3.577	3999540	Nonane	128.157	000111-84-2
4.274	1153833	1,9-Decadiene	138.141	001647-16-1
4.342	13712916	1-Decene	140.157	000872-05-9
4.410	3612347	Decane	142.172	000124-18-5
5.165	1032489	1,10-Undecadiene	152.157	013688-67-0
5.233	9383561	Cyclopropane, 1-methyl-2-pentyl-	126.141	041977-37-1
5.307	4486711	Undecane	156.188	001120-21-4
6.072	1089075	1,11-Dodecadiene	166.172	005876-87-9
6.135	5851250	1-Dodecene	168.188	000112-41-4
6.203	4530114	Dodecane	170.203	000112-40-3
6.963	776054	1,13-Tetradecadiene	194.203	021964-49-8
7.016	4127532	1-Tridecene	182.203	002437-56-1
7.073	3306911	Tridecane	184.219	000629-50-5
7.859	3621011	2-Tetradecene, (E)-	196.219	035953-54-9
7.907	2582932	Tetradecane	198.235	000629-59-4
8.662	1598319	1-Pentadecene	210.235	013360-61-7
8.703	1706322	Pentadecane	212.250	000629-62-9
9.453	1362194	1-Decanol, 2-octyl-	270.292	045235-48-1
10.203	410585	1-Decanol, 2-hexyl-	242.261	002425-77-6

Table S12. Identified substances after pyrolysis (500 °C) of sample 12.

RT (min)	Area (Ab*s)	Hit Name	Mol Weight (amu)	CAS Number
1.899	15793435	1,3-Butadiene	54.047	000106-99-0
2.041	13942496	2-Propenenitrile	53.027	000107-13-1
2.298	10342312	Benzene	78.047	000071-43-2
2.754	164800549	Toluene	92.063	000108-88-3
3.116	23651592	Cyclohexene, 4-ethenyl-	108.094	000100-40-3
3.325	31308921	Benzene, 1,3-dimethyl-	106.078	000108-38-3
3.619	2422495166	Styrene	104.063	000100-42-5
3.834	1469926	1,3,5,7-Cyclooctatetraene	104.063	000629-20-9
4.012	9768539	Benzene, 2-propenyl-	118.078	000300-57-2
4.075	3301278	1,3,5-Cycloheptatriene, 7-ethyl-	120.094	017634-51-4
4.316	67328304	.alpha.-Methylstyrene	118.078	000098-83-9
4.405	3861713	(Z)-1-Phenylpropene	118.078	000766-90-5
4.714	12138494	Benzene, 1-propenyl-	118.078	000637-50-3
4.861	3944921	Benzene, 3-butenyl-	132.094	000768-56-9

4.908	2252128	Indene	116.063	000095-13-6
4.982	4937939	Benzene, (1-methylenepropyl)-	132.094	002039-93-2
5.301	574389	Benzene, (2-methyl-3-butenyl)-	146.110	001647-06-9
5.758	2684427	Benzene, (1-methylenebutyl)-	146.110	005676-32-4
5.883	4554356	Bicyclo[4.2.0]oct-1-ene, 7-exo-ethenyl-	134.110	1000142-18-2
5.957	2106957	Benzene, 3-pentenyl-	146.110	001745-16-0
6.104	2163323	Cyclohexane, 1,5-diethenyl-3-methyl-2-methylene-, (1.alpha.,3.alpha.,5.alpha.)-	162.141	074742-35-1
6.544	3257288	Benzene, 1-hexynyl-	158.110	001129-65-3
7.477	649602	Benzene, 3-cyclohexen-1-yl-	158.110	004994-16-5
9.243	2848125	Benzene, 1,1'-(1-methyl-1,2-ethanediyl)bis-	196.125	005814-85-7
10.512	11004363	n.i.	n.i.	n.i.
